# Self-assembly of amphiphilic truncated cones to form hollow nanovesicles†

**DOI:** 10.1039/c8ra01100a

**Published:** 2018-04-10

**Authors:** Yali Wang, Xuehao He

**Affiliations:** Department of Chemistry, School of Science, Tianjin University Tianjin 300350 China xhhe@tju.edu.cn; Demonstration Centre for Experimental Chemistry & Chemical Engineering Education, Tianjin University Tianjin 300350 China; National Virtual Simulation Experimental Teaching Centre for Chemistry & Chemical Engineering Education, Tianjin University Tianjin 300350 China

## Abstract

To mimic the unique properties of capsid (protein shell of a virus), we performed Brownian dynamics simulations of the self-assembly of amphiphilic truncated cone particles with anisotropic interactions. The particle shape of a truncated cone in our simulations depended on the cone angle *θ*, truncated height *h*_c_ and particle type (A_*x*_B_*y*_ and B_*x*_A_*y*_B_*z*_). The hydrophobic A moieties and hydrophilic B moieties are responsible for attractive and repulsive interactions, respectively. By varying the particle shape, truncated cones can assemble into hollow and vesicle-like clusters with a specific cluster size *N*. To assemble into hollow vesicles, the truncated height *h*_c_ must be below a critical value. When *h*_c_ exceeds this critical value, malformation will occur. The dynamics shows that the vesicle formation occurs in three stages: initially the growth is slow, then rapid, and finally it slows down. The truncated height *h*_c_ has a stronger impact on the growth kinetics than the cone angle *θ* or the particle type. We explored how the cluster packing depended on the cooling rate and particle number as well as discussing the relationship between the cluster geometry and the interparticle interactions. Further, we also discuss possible methods to experimentally prepare the truncated cones. The results of our work deepen our understanding of the self-assembly behavior of truncated cones and our results will aid the effective design of particle building blocks for novel nanostructures.

## Abstract 2

To mimic the unique properties of capsid (protein shell of a virus), we performed Brownian dynamics simulations of the self-assembly of amphiphilic truncated cone particles with anisotropic interactions.

## Graphical abstract

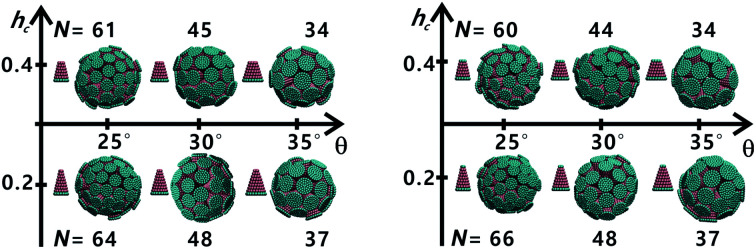

## 1. Introduction

Nanoparticle self-assembly imitates the organization of atoms or molecules into crystals or supramolecular structures^1^ and it has attracted intense research interest owing to its usefulness for the design and fabrication of novel nanostructured materials.^2–4^ In the nanoparticle self-assembly process the particle shape plays a key role and is particularly important for exploring new nanostructures and their functionalities.^5,6^ In addition to the shape of building blocks, the interactions between individual subunits is also vital for the assembly process. The subunit interactions are mainly determined by the shape, surface properties, and inherent physical properties (*e.g.*, charge, polarizability, dipole, and mass) of the subunits. A key aim in the field of nanoparticle self-assembly is the exploitation of highly specific and directional interactions in combination with an anisotropic building block shape to achieve precisely ordered structures to broaden the range of applications.

In biological systems, peptides and proteins have the ability to assemble into advanced structures.^7^ Ionic self-complementary peptides can form nanofibers^8^ and short peptides can self-assemble into semiflexible nanotapes,^9^ while surfactant-like peptides can form nanotubes, nanovesicles^10,11^ and supramolecular structures.^12,13^ Recent experiments have demonstrated that recombinant fusion proteins can form an amphiphilic complex to further self-assemble into globular protein vesicles by fine turning the protein concentration or temperature;^14^ and both vesicle size and vesicle geometry can be well-controlled. Beside vesicles, solid particles and hierarchical supraparticles can also be formed through rational protein design.^15^ Peptides and proteins were treated as cone-like molecules in above mentioned studies, which inspired us to understand their assembly mechanism in more depth and helped us to more effectively design novel building blocks for assembly. With regard to non-biological systems, several assembly systems such as amphiphilic block copolymers,^16^ metal-polymer amphiphiles^17^ and patchy colloidal dumbbells^18,19^ have often been regarded as a cone-like molecules and they can assemble into various structures (*e.g.*, spherical micelles, cylindrical micelles and vesicles) owing to the inherent curvature of building blocks. Cone-like particles or molecules are typical building blocks for assembly and they generally have a nonsymmetrical shape with regard to their two ends. Although much experimental progress has been made in the self-assembly of cone-shaped molecules and particles, some issues such as assembly pathways and the influence of building blocks' parameters including shape and interparticle interaction are not yet exhaustively understood.^20^

Compared with experimental methods, computer simulations are powerful tools for exploring the self-assembly of specific-shaped particles, although it is difficult to simultaneously consider the particle shapes and interparticle interactions.^21–24^ Because of computational limitations, the direct calculation of nanoparticle self-assembly using an all-atom model is not feasible; instead one must consider coarse-grained model systems. The widely adopted coarse-grained model is the Kern–Frenkel model^25^ of patchy spherical particles with short-ranged directional attraction. The whole particle is treated as a “bead” and the patch is either attached to the bead's surface or part of it. Models of this class are formulated in terms of spherical particles with symmetric, regular patches because the interparticle attractions are restricted to orientations in which two patches face each other. In Kern–Frenkel model, the anisotropy of interactions strongly depends on the number of patches and their size.^25–27^ Another general model with anisotropic interactions was proposed by Zhang *et al.*,^28^ who used a more complex coarse-grained model in which the whole particle was constructed from a large number of coarse-grained beads. This coarse-grained model greatly reduced the overall number of interactions that must be calculated compared with an all-atom model. The same or complementary beads within the patches interacted with either attractive or repulsive interactions. This coarse-grained model is capable of describing particles and patches with irregular shape and size and it can easily be modified to study the assembly of nonspherical particles.^29,30^ In this work, the simplified coarse-grained model was used to characterize the shape of the truncated cone, and a truncated cone included two types of coarse-grained beads to separately represent hydrophobicity and hydrophilicity.

Gaining a quantitative description of a particle's shape and interparticle interactions is a critical and challenging task in assembly simulation of nonspherical particles. Recently, we developed an effective simulation method for the assembly of nonspherical particle with the help of tabulated potential technology.^30^ Utilizing this model, the self-assembly of two kinds of cone-like particles with a series of cone angles was studied, and various spherical structures with a precise cluster size were formed.^24^ In that system it was found that clusters began to form simultaneously and the formed clusters aggregated uncontrollably. Those formed clusters were not hollow structures, which considerably prohibits their potential applications,^13,31^ especially for drug delivery which requires dispersed hollow clusters in solutions.^13^ In this study, a truncated cone with an amphiphilic surface was designed. Meanwhile, purely repulsive interactions were introduced for hydrophilic coarse-grained beads, which imitates the hydrophilic parts of amphiphilic molecules and it can avoid the cluster–cluster aggregation. The aim of this work was to reveal how the shapes of truncated cone particles affect the self-assembly process and to explore the relationship between complex particle shapes and simple cluster geometry. We employed a quantitative Brownian dynamics (BD) simulation framework for nonspherical particles that was proposed in our prior work.^30^ This BD framework applied the tabulated potential and the interpolation techniques, and it has advantages of being highly efficient at calculating anisotropic interparticle interactions. This force field between two interacting assembly particles has been calculated before the BD simulations begin. This BD simulation framework not only ensures the precise description of the particle shape and anisotropic interparticle interactions, and it also significantly improves the simulation's efficiency.

Herein, we employ the novel BD simulation framework to study the self-assembly of a series of truncated cones with different particle shapes. General descriptions involved in coarse-grained model of the truncated cones and detailed information on the simulation method are introduced in section 2. The influence of the particle shape on assembled structures, cluster size, cluster geometry, aggregation temperature and cluster growth kinetics are discussed in section 3; the dependence of cooling rate and particle number on the self-assembly and the relationship between interparticle interaction and cluster morphology are also discussed in this section. Furthermore, possible synthesis methods for cone-like particles are suggested, and finally a brief summary of this work is presented in the conclusion section.

## 2. Model and method

**Fig. 1:**
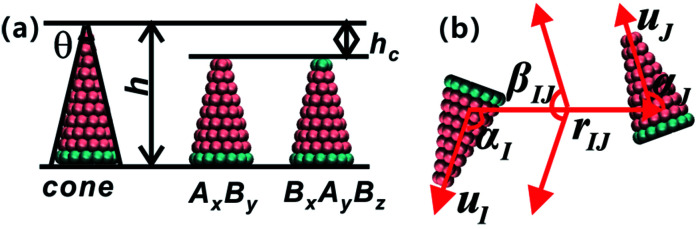
(a) Illustration of the coarse-grained model of truncated cones. Here, A and B coarse-grained bead are indicated by pink and cyan colours, respectively. The left one is a complete cone with height *h* and cone angle *θ*, and the middle A_*x*_B_*y*_ type particle is evolved from the left complete cone by subtracting a constant height *h*_c_. The right B_*x*_A_*y*_B_*z*_ type particle stems from the replacement A beads of the “tail” layer with B beads. (b) The schematic diagram of particle orientation. Particle orientation can be described as a set of angles (*α*_I_, *α*_J_, *β*_IJ_), orientational angles *α*_I_, *α*_J_ and dihedral angle *β*_IJ_. *u*_I_ and *u*_J_ are unit orientational vectors and *r*_IJ_ is the centroid–centroid vector between particles I and J.

The coarse-grained model of truncated cones is illustrated in Fig. 1a. Particles are composed of two types of spherical coarse-grained beads. Pink A beads and cyan B beads indicate hydrophobicity and hydrophilicity, respectively. Truncation was used for the particle shape transformation. Truncated cones were designed by cutting complete cones to a certain height, and the truncated height is defined as *h*_c_. The full height *h* of a cone was set to 1.0 nm, and the spacing of adjacent beads and layers within a single particle is 0.1 nm. Present simulation only shows the general rules of self-assembly of the truncated cones. The particle size used don't correspond to the real system. A particle's shape is characterized by the two-dimensional cone angle *θ* (*i.e.*, *θ* = 25°, 30°, 35°), truncated height *h*_c_ (*e.g.*, *h*_c_ = 0.2, 0.4, 0.5, 0.6, 0.7 nm) and particle type. Two particle types, namely A_*x*_B_*y*_ and B_*x*_A_*y*_B_*z*_, were constructed. The subscript *x*, *y*, *z* represents the number of coarse-grained beads of type A and B. The bottom layer in an A_*x*_B_*y*_ type particle consists of B beads. Unlike in an A_*x*_B_*y*_ type, both the top and bottom layers of a B_*x*_A_*y*_B_*z*_ type particle are composed of B beads. We studied whether replacing one layer of B beads to the “tail” end would affect the cluster size and cluster growth kinetics. The length and overall hydrophobicity of a particle can be fine-tuned by modifying the number of A coarse-grained beads. The hydrophobic tails of the particles contained an increasing number of A beads and thus had higher hydrophobicity. The number of coarse-grained beads in the various truncated cones is listed in Table S1.†

For hydrophobic A components, the interaction between A beads is Lennard-Jones potential and that is *U*_*ij*_ = 4*ε*_AA_[(*σ*/*r*_*ij*_)^12^ − (*σ*/*r*_*ij*_)^6^], while for the hydrophilic B components purely repulsive interactions are used to reflect its hydrophilicity, *U*_*ij*_ = *ε*_BB_(*σ*/*r*_*ij*_)^5^. The interaction between two unlike A and B beads is the arithmetic mean of like beads, which is
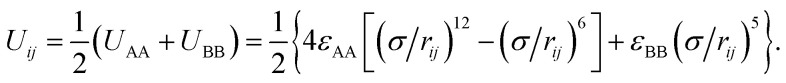


Here, *ε*_AA_ and *ε*_BB_ are interaction strength parameters, *σ* is interaction range, which is equal to the diameter of a coarse-grained bead (*σ* = *σ*_AA_ = *σ*_BB_ = *σ*_AB_ = 0.1 nm), and *r*_*ij*_ is the distance between the centre of two interacting beads. The interaction strength parameters are defined to ensure that the A beads are strongly hydrophobic and the B beads are weakly hydrophilic (*ε*_AA_ = 2.0 KJ mol^−1^ and *ε*_BB_ = 0.2 KJ mol^−1^).

According to the interactions used above, the interaction between two truncated cones (I and J) is the sum of the interactions between coarse-grained beads within the truncated cones,1
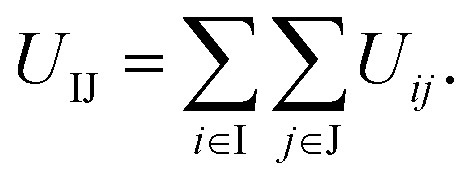


Here, the truncated cones are treated as rigid bodies and the interparticle interactions are directionally dependent. The computation cost for *U*_IJ_ is high in every simulation step. Instead of this, we used an alternative anisotropic potential, given by2
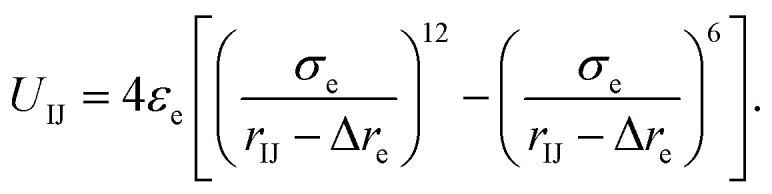


The strength parameter *ε*_e_, range parameter *σ*_e_ and shape parameter *r*_e_ were calculated using a numerical matching method.^30^ These three parameters are orientation-dependent and the orientation between two particles is represented with three angles *α*_I_ ∈ [0,π], *α*_J_ ∈ [0,π] and *β*_IJ_ ∈ [0,π], as shown in Fig. 1b. Each orientation angle is divided into 99 equal parts when calculating the tabulated potentials.

The three interaction parameters in eqn (2) can be solved only when eqn (1) yields a potential curve with a negative local minimum.^30^ However, in the present system *U*_IJ_ maybe positive without local minimum due to the introduction of the purely repulsive interaction between B beads. The potential fitting method must be modified accordingly, and thus we added an additional attractive interaction *U*^add^ = −1.0/(*r*_IJ_ − 0.1) to *U*_IJ_ in eqn (1) in the potential calculation. The additional attraction *U*^add^ can lead to the existence of a negative local minimum in new potential curves at all orientation directions. Consequently, the *U*_IJ_ in eqn (1) can be well fitted with the anisotropic potential in eqn (2). Note that the additional attractive potential *U*^add^ is excluded in the calculation of translation force and torque in BD simulations. Examples of potential calculation are shown in Fig. S1.†

Because there is a short-ranged interparticle interaction expressed in eqn (2), and to further improve the potential calculation efficiency and to increase the calculation convergence rate, a soft-core potential is introduced when *r*_IJ_ < *r*_m_ and the potential calculation is paused at *r*_IJ_ > *r*_m_. Thus, the final form of the anisotropic interparticle interactions can be written as3
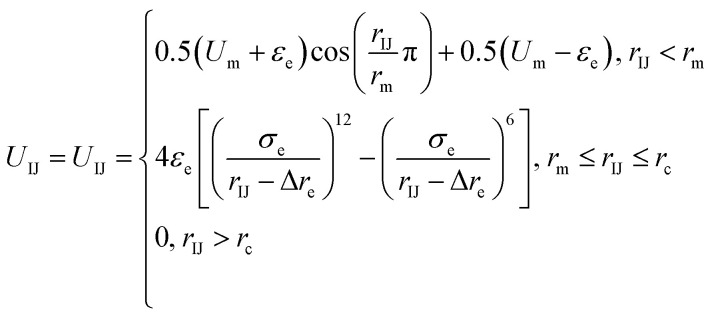
where *r*_m_ = 2^1/6^*σ*_e_ + Δ*r*_e_ is the equilibrium distance to the minimum potential, *r*_c_ = 3.0 nm is the cutoff distance, and *U*_IJ_ is equal to *U*_m_ at *r*_IJ_ = 0 nm (*U*_m_ = 500 KJ mol^−1^).

In present coarse-grained model, we only consider the global interactions between particles is attractive or repulsive. Such interaction does not correspond to any real interaction type. In our BD simulation, hydrodynamic interactions is also ignored and the random force represents the randomly fluctuating force exerted on a particle by the surrounding fluid. An implicit solvent model was used here. Our tabulated potential method has an advantage in quantitatively describing specific particle shapes and anisotropic interparticle interactions, unlike other methods such as the patch model which presents anisotropy *via* the patch size and patch position. The direction-dependent interactions between particles arise from all attractive and/or repulsive beads that make up the particles, not from the “atoms” within the patches. This potential computation method is not only suitable for symmetric, regular particle shape but in principle also for arbitrary shapes. In a BD simulation, the computation cost is entirely unrelated to the number of coarse-grained beads within a particle because the interparticle interactions in BD simulations are calculated with interpolation methods based on tabulated potentials.

To study the self-assembly of truncated cones, a rigid body BD simulation in the canonical ensemble was applied. The simulated box was cubic with a box side *l*; periodic boundary conditions were used. At each BD step, each particle attempts to move and rotate according to the following equations of motion:4
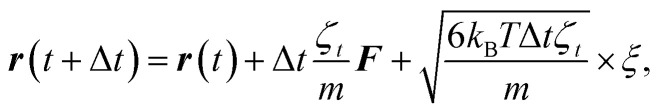
5
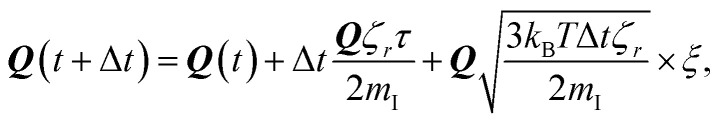
where *m* = 1.33 u is the mass of a particle; *m*_I_ = 1.01 u nm^−2^ rad^−2^ is the moment of inertia of a particle; *ζ*_*t*_ = 0.005 ps and *ζ*_*r*_ = 0.005 ps are the transaction and rotation friction constants, respectively; the time step Δ*t* = 0.01 ps is used to integrate the discretized equations of motion; *k*_B_ is the Boltzmann constant; *ξ* is the standard Gaussian distributed noise; and the translation force *F =* −(*E*_*r+*d*r*_ − *E*_*r*−d*r*_)/2d*r* and the torque *τ* = −(*E*_*θ*+d*θ*_ − *E*_*θ*−d*θ*_)/2d*θ* are both calculated from the potential energy difference between the conformations before and after the trial motion by a small amount of translational displacement (1.0 × 10^−5^ nm) and rotational displacement (1.0 × 10^−5^ rad).

All BD simulations were cooled from a high-temperature, initially disorder conformation of *M* particles to a target temperature with stable structures. The low target temperature of each simulation was chosen to allow sufficient time for the particles to assemble into stable structures with unnoticeable change of the system's total energy (the annealing temperature range for each simulation is listed in Table S1†). Unless otherwise stated, an annealing rate, 2.0 × 10^5^ step per K, was applied to avoid kinetically trapped structures. This cooling rate was proved to be affordably slow and yet also fast and efficient enough, compared with using 1.0 × 10^5^ step per K or 3.0 × 10^5^ step per K to produce stable clusters. The specific heat capacity *C*_v_ was used to monitor changes of the system's energy. Our studies were restricted to the dilute solution regime, *i.e.*, *M* = 100 particles were dispersed in a cubic simulation box with a box side *l* = 10 nm, mainly to reduce the computational time.

## 3. Results

### 3.1 Truncated cone shapes and assembled structures

**Fig. 2:**
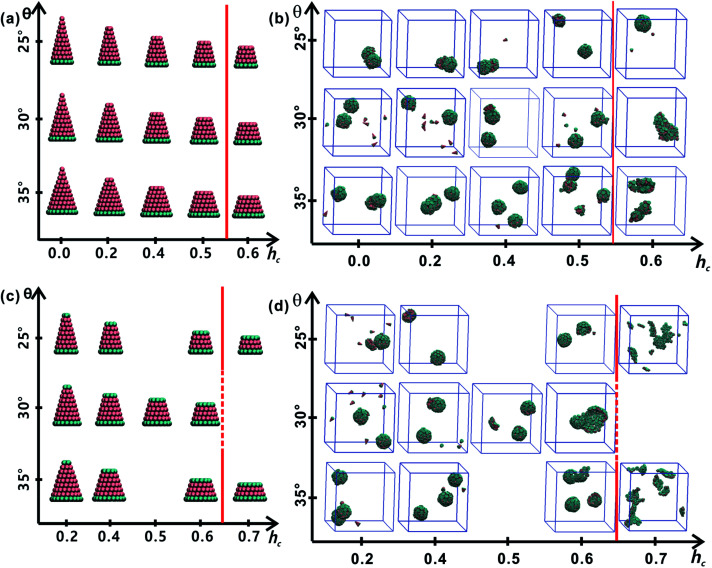
Summary of particle shapes and self-assembled structures of each kind of particle shape. Particle shapes of A_*x*_B_*y*_ type and B_*x*_A_*y*_B_*z*_ type are respectively, shown in (a) and (c). The assembled structures generated from particles of A_*x*_B_*y*_ type particles is exhibited in (b) and those of B_*x*_A_*y*_B_*z*_ type particles are listed in (d). The red lines in (b and d) indicate the boundaries of regular (left) and malformed (right) structures.

All truncated cone-shaped particles studied in this work are listed in Fig. 2a and c. The self-assembly process of twenty-four truncated cones and three complete cones were simulated using the annealing method. The initial state of system was obtained at a high enough temperature where all particles are randomly distributed in solution owing to thermal motion. As the system temperature gradually decrease, truncated cones strive for the maximum number of nearest neighbours and they are bound tightly to each other. Meanwhile, repulsive interactions between the ends of the truncated cones propel the assembled particles into curved, closed structures. Fig. 2 shows the final assembled structures generated in the simulations for each particle shape in simulations. Notably, most of the truncated cones self-assemble into discrete vesicle-like structures. As expected, the geometric arrangement of the cluster mainly depends on building block shape and interparticle interactions. The assembled clusters were hollow, and for those constructed with A_*x*_B_*y*_ particles, the inner wall was composed of A component and was thus hydrophobic; for B_*x*_A_*y*_B_*z*_ type particles, B component made up the hydrophilic cavity wall.

**Fig. 3:**
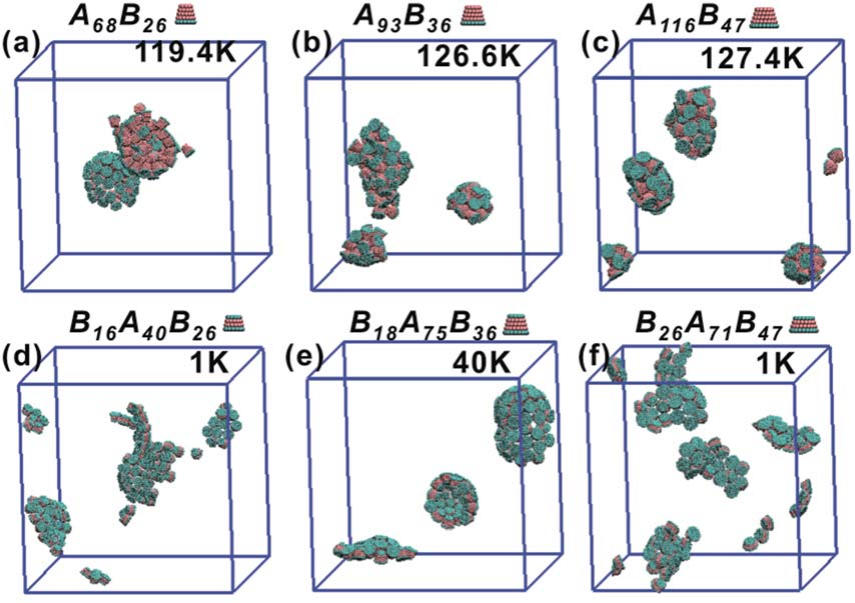
A classification of the malformation as a function of particle shape is shown, (a) for A_*x*_B_*y*_ type particles at *θ* = 25° and *h*_c_ = 0.6, (b) for A_*x*_B_*y*_ type particles at *θ* = 30° and *h*_c_ = 0.6, (c) for A_*x*_B_*y*_ type particles at *θ* = 35° and *h*_c_ = 0.6, (d) for B_*x*_A_*y*_B_*z*_ type particles at *θ* = 25° and *h*_c_ = 0.7, (e) for B_*x*_A_*y*_B_*z*_ type particles at *θ* = 30° and *h*_c_ = 0.6 and (f) for B_*x*_A_*y*_B_*z*_ type particles at *θ* = 35° and *h*_c_ = 0.7. The annealing rate of each system is 2 × 10^5^ step per K.

As the truncated height *h*_c_ increased, the inner diameter of the hollow structures also increased. However, the increase of *h*_c_ was unfavourable for forming ordered clusters. When the truncated height *h*_c_ was very large, order packing was difficult to achieve and malformations were observed. There exists a critical border for each set of truncated cone at the same *θ* to assemble into vesicle (see Fig. 2). For A_*x*_B_*y*_ particles, the critical value of *h*_c_ is 0.5 (Fig. 2b) and for B_*x*_A_*y*_B_*z*_ particles the critical values of *h*_c_ is 0.6, except for *θ* = 30° (Fig. 2d). The disparity of the critical value of *h*_c_ at *θ* = 30° was caused by the fluctuation of simulation data. A truncated cone was evolved from a complete cone by cutting several layers from its top tip. It is not a smooth evolution of particle shape, which may lead to the error of the statistical data in a certain particle shape. The source of the malformations of A_68_B_26_ particles at *θ* = 25°and *h*_c_ = 0.6 was the development of small local errors in the growing micelles. The clusters continue to grow when there is a malformation, and finally a malformed structure is generated and it is similar to a correct vesicle (Fig. 3a). For A_93_B_36_ and A_116_B_47_ particles at *θ* = 30°, 35°and *h*_c_ = 0.6, the source of the malformation is the inability of malformed clusters to correct themselves after the occurrence of a malformation (Fig. 3b and c). While for B_*x*_A_*y*_B_*z*_ particles, the origin of malformations of B_18_A_75_B_36_ particles at *θ* = 30° and *h*_c_ = 0.6 is interactions of incomplete clusters; two incomplete clusters can connect together into a single malformed structure and the malformed structure can continue to attract free particles (Fig. 3e). For B_16_A_40_B_26_ and B_26_A_71_B_47_ particles at *θ* = 25°, 35° and *h*_c_ = 0.7 the interparticle attractive interactions are not sufficient to counterbalance the repulsive volume effect in the assembly when *h*_c_ is large enough (Fig. 3d and f).

### 3.2 Cluster sizes and cluster geometries

**Fig. 4:**
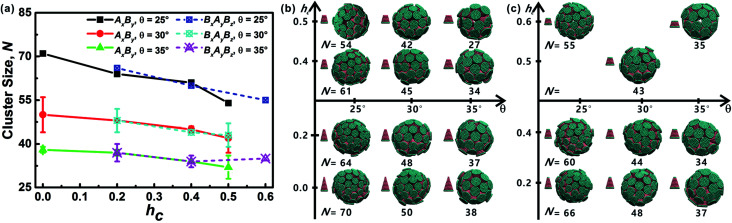
(a) Cluster size *N* of each kind of particle shape. The error bar indicates the size difference of two formed clusters. Note that for particles at *θ* = 25° there is only one complete cluster formed, and thus there is no error bar. (b and c) Cluster geometries with corresponding particle shape and cluster size *N* are shown. Cluster morphology for both A_*x*_B_*y*_ and B_*x*_A_*y*_B_*z*_ type particles is sphere-like and hollow, similar to vesicle-like structures formed by amphiphilic molecules.

As mentioned above, truncated cones can assemble into a series of unique vesicle-like clusters with a specific cluster size *N*; the cluster size is defined as the number of particles that form a single cluster. Cluster size of each assembled aggregates was calculated. The cluster size *N* is shown in Fig. 4a as a function of the particle shape. It was found that the cluster size *N* increases with decreasing cone angle *θ*, and the cluster size *N* decreases gradually with increasing *h*_c_. The downward trend is most pronounced at *θ* = 25°. But the dependence of the cluster size *N* on particle type is not significant. These results indicate that cone angle *θ* and truncated height *h*_c_, rather than particle type, determine the cluster size *N* of the assembled vesicles and the most critical factor is the cone angle *θ*. The conclusion in this work is in agreement with the conclusions of previous studies^21,22,24,32^ and the previous conclusion is that the cluster size of cone-shaped particles increases as the cone angle decreases.

Cluster geometries with their corresponding particle shapes and cluster size are shown in Fig. 4b and c. There are chemical similarities between amphiphilic molecules and the truncated cones, because both amphiphilic molecules and the truncated cones consist of hydrophobic and hydrophilic parts. The hydrophobic parts of truncated cones pack closely to form hollow and spheroid structure, and the formed structures resemble vesicles comprised of amphiphilic molecules. However, the spheroid structure does not agree with the prediction results of the “critical packing parameter” (CPP).^33^ Truncated cones with 1/3 < CPP ≤ 1/2 should assemble into cylinders. The difference between the CPP expected structures and the actual assembled structures was also been observed by other groups.^21,22,32^ Tsonchev *et al.*^32^ argued that the relationship between CPP values and the aggregation shapes is not unique and the CPP method should merely be reviewed as a guide. Spherical structures are energetically preferred because there is always a higher number density of cones or truncated cones for spherical structures than for cylinders.^32^ Thus, it is clear that the problem is to find the densest structure that can be formed using truncated cones. Additionally, Chen *et al.*^21^ suggested that the disparity may be limited to rigid cone systems, because the CPP method is suitable for the soft amphiphiles.

In this work, the hydrophobic A components are responsible for hydrophobic attractive interactions and the attractive interactions lead to effective self-assembly, and the hydrophilic “heads” (*i.e.*, B components) only interact with repulsive interactions and the repulsive interactions are unfavourable to effective self-assembly. When the truncation height *h*_c_ is larger than the critical *h*_c_ illustrated in Fig. 2, the hydrophilic area of each particle will obviously increase; the result is that the repulsive force between particles also increases significantly, which is not favourable for the effective self-assembly of spherical structures because the attractions between particles are not large enough to inhibit malformed self-assembly. Thus, the densest structures ought to be the results of the balance between the hydrophobic attractive interactions and hydrophilic repulsive interactions. Here, entropic effects were not considered because our previous work^24^ suggested that the contributions of entropic effect on self-assembly can be omitted, because the entropic effect is very small compared with the interparticle interactions. Furthermore, truncated cones are regarded as rigid bodies and all simulations were carried out in dilute systems (particle number density of 0.1 nm^−3^). The CPP packing rule is actually more suitable for predicting the behaviour of amphiphiles with soft tails,^17,34^ and for soft amphiphiles by increasing the tail length, the cluster geometries can transform from spherical micelles to cylinders to bilayers. It is obvious that our current model of truncated cones is not suitable for the CPP packing rule because our hydrophobic “tails” are rigid.

In addition, it is instructive to compare the packing of our truncated cones to the virus capsid (protein shell of a virus) assembly. Most studies of virus capsid assembly process usually impose symmetry constraint, *e.g.*, spherical or icosahedral,^35,36^ to the cluster geometry before assembly. Thus, the problem is limited to an optimal packing for a few subunits constrained to the surface of a spherical or icosahedral shell.^37,38^ Our simulation have no special approximation or limitation with regard to the assembly of the truncated cones. We found some cases for which the assembled vesicles (cluster size *N* = 34, *N* = 42 and *N* = 70) are very similar to several virus capsid protein, *i.e.*, *cowpea chlorotic mottle virus*^39^ capsid with *T* = 3 Caspar and Klug (CK) structure (*N* = 32), a NωV virus^40^ capsid with *T* = 4 CK structure (*N* = 42) and a *polyoma virus*^41^ capsid with *T* = 7 CK structure (*N* = 72).

### 3.3 Self-assembly process of truncated cones

The specific heat capacity *C*_v_ was chosen as the second criterion to estimate the assembly process and it can accurately reflect the energy fluctuation in assembly process. The *C*_v_ can be calculated with*C*_v_ = ∂*E*/∂*T* = 1/*k*_B_*T*(〈*E*^2^〉 − 〈*E*〉^2^).

**Fig. 5:**
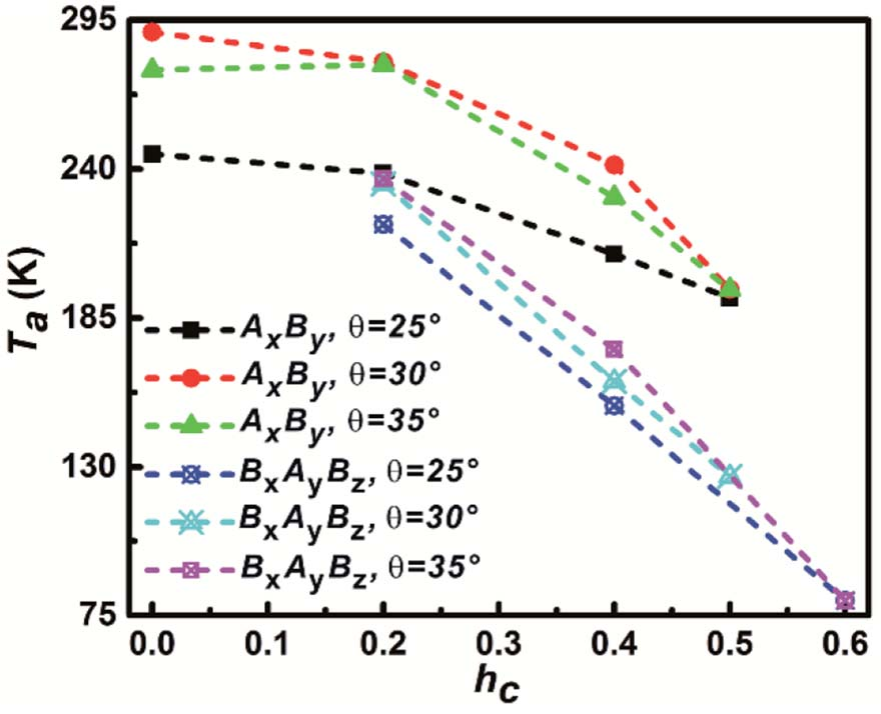
The dependence of aggregation temperature *T*_a_ on the particle shape. The *T*_a_ is defined as the temperature corresponding to the first specific capacity heat *C*_v_ peak.

The temperature that corresponds to the first maximum *C*_v_ value is defined as aggregation temperature, *T*_a_ and the influence of particle shape on *T*_a_ is shown in Fig. 5. It can be seen that the *T*_a_ of A_*x*_B_*y*_ particles is higher than that of B_*x*_A_*y*_B_*z*_ particles at the same *θ* and *h*_c_. At the same *θ*, *T*_a_ decreases with increasing *h*_c_; furthermore, the decrease of *T*_a_ for the B_*x*_A_*y*_B_*z*_ particles occurs at an obviously higher rate than for A_*x*_B_*y*_ particles. For the same *h*_c_, *T*_a_ increases with increasing *θ* for both A_*x*_B_*y*_ and B_*x*_A_*y*_B_*z*_ particles. All these phenomena can be explained by considering the differences in the particle hydrophobicity and hydrophilicity. The stronger the hydrophobicity, the better the effective self-assembly, and the more effective self-assembly leads to a higher *T*_a_.

**Fig. 6:**
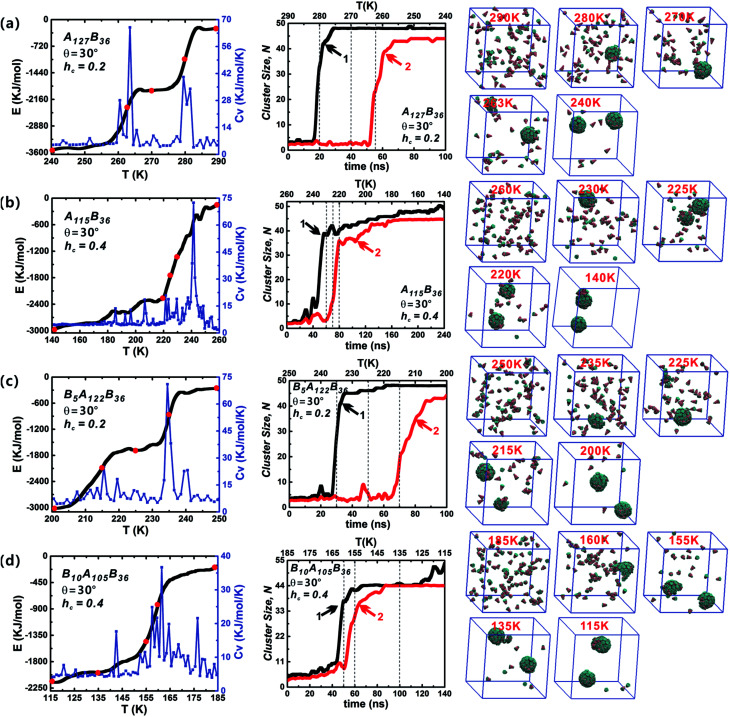
The energy *E*, specific capacity heat *C*_v_ and cluster growth kinetics of A_*x*_B_*y*_ and B_*x*_A_*y*_B_*z*_ type particles at *θ* = 30° and *h*_c_ = 0.2, 0.4. The snapshots of self-assembled intermediate configurations at several typical temperatures (indicated by red points on energy evolution curves) are also shown. The specific capacity heat *C*_v_ reflects the system energy fluctuation in the self-assembly process. The dash lines on cluster growth kinetics correspond to those red temperature points on energy evolution curves. The final assembled structures mainly include two complete clusters, and the black and red curves correspond to the growth of the first and second cluster, respectively.

Both the success of forming discrete clusters and the growth kinetics depend delicately on the aggregation energies of the free truncated cones. The influence of *h*_c_ on the self-assembly process was investigated for A_*x*_B_*y*_ and B_*x*_A_*y*_B_*z*_ particles at *θ* = 30°, as shown in Fig. 6 (the date of other particle shapes is shown in Fig. S2–S4†). The sharp changes in energy are reflected in the *C*_v_ curves. At *h*_c_ = 0.2, the decline in energy is stairs-like with obvious plateaus. When *h*_c_ is 0.4 and 0.5, the system' total energy decreases continuously and the stairs-like downward trend of energy is no longer obvious; accordingly, there is only one distinct sharp peak in the *C*_v_ curve. Compared with the effects of particle type and cone angle *θ*, the truncated height *h*_c_ has a strong impact on system energy fluctuations. With the increase of *h*_c_, the hydrophobicity of particles weakens and the hydrophilicity of particles becomes stronger, that is, the repulsive interactions between particles increase with the increment of *h*_c_, which is not favourable to minimizing the system energy, and thus the system rapidly enters the growth stage of second cluster. The typical configurations of the particles at various temperatures are shown in Fig. 6. Initially, particles are randomly distributed, followed by the formation of curved partial structures, and then a well-packed cluster is generated; thereafter, the remaining free particles begin to grow to form the second cluster until its completion. In the whole assembly process, clusters are formed one-by-one, not together. The reason is that when the temperature decreases to a critical value, a stable nucleus starts to create and particles aggregate into the nucleus till a complete vesicle forms. When a vesicle is formed, the concentration of the free particles in system decreases. It becomes difficult to form a new stable nucleus. Only when the temperature is further decreased, the second stable nucleus can be formed. So, clusters are formed one-by-one in such annealing process.

The role of particle type in the assembly kinetics at *θ* = 30° was also studied (Fig. 6, and the data for other shapes is shown in Fig. S2–S4†). Three distinct periods are visible: (1) initial lag, closely followed by (2) rapid growth, and finally (3) growth slow-down. Such growth kinetics traits resemble classical nucleation and growth mechanism of virus capsids.^42^ Stage (1) clearly defines the time range in which nucleation occurs, *i.e.*, in which particles diffuse and collide freely, thus producing the critical growth nucleus. Once the growth nucleus has been formed, the system enters a period of fast growth and the number of free particle decreases rapidly until a robust cluster has been formed. For the same *h*_c_ value, the role of particle type in the assembly kinetics is not obvious. However, increasing *h*_c_ has a significant impact on the growth kinetics: a distinct lag period for the growth of the second vesicle is observable at *h*_c_ = 0.2. But this distinct lag stage vanishes when *h*_c_ is 0.4 or 0.5, and the second clusters (red curves) begin to grow immediately once the generation of the first clusters (black curves) is complete. In addition, it was found that there are some fluctuations in the cluster size, and these fluctuations indicate the merger of clusters. The merger of clusters will creates oversize misassembled structures, and they are usually generated from complete vesicles and partial vesicles. Fortunately, these oversize misassembled structures can be corrected immediately and they are different to the malformation mentioned in Fig. 3. In brief, varying particle shape, especially *h*_c_, changes the interparticle interaction significantly and plays an important role in the vesicle growth kinetics.

**Fig. 7:**
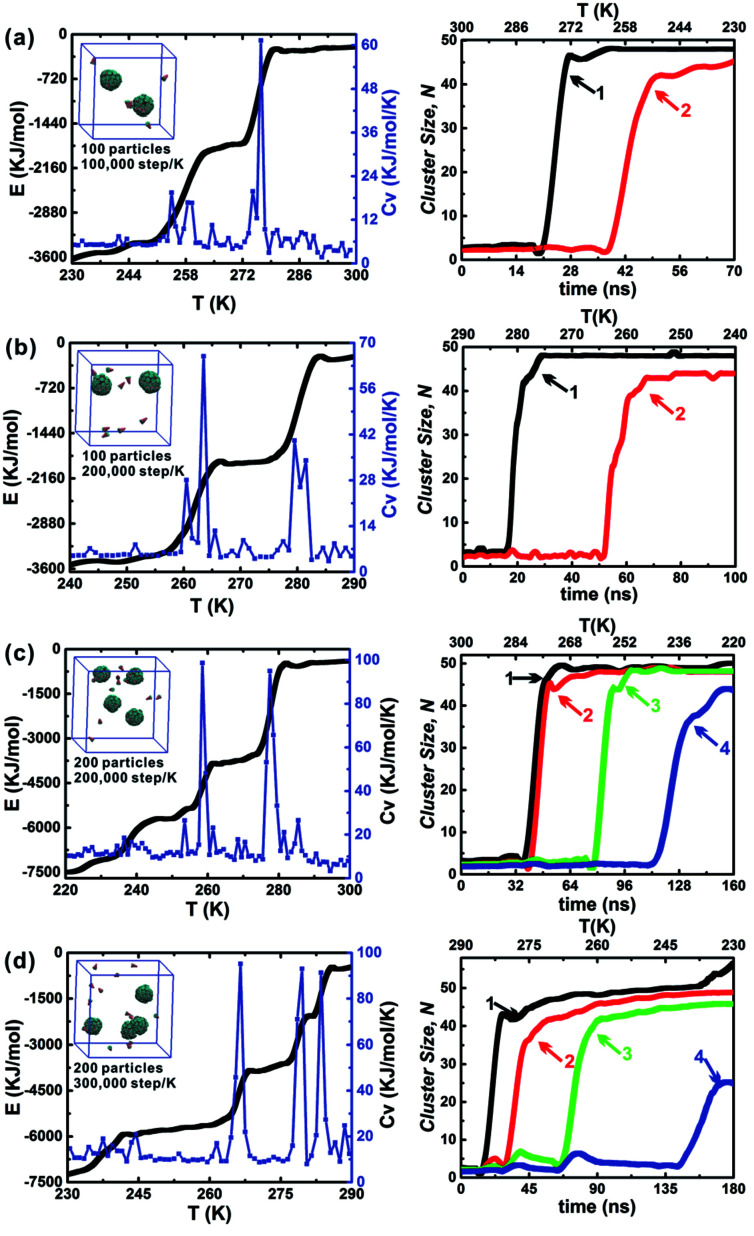
Dependence of annealing rate and particle number on the self-assembly of A_*x*_B_*y*_ type particles at *θ* = 30° and *h*_c_ = 0.2. The final assembled configurations of each system are shown, which can be seen insets in the energy evolution curves; the right column is the corresponding cluster growth kinetics. There are three annealing rates, 1.0 × 10^5^ step per K, 2.0 × 10^5^ step per K and 3.0 × 10^5^ step per K. 100 particles is in a 10 nm cubic simulation box and 200 particles is in a 12.6 nm cubic simulation box, which ensures the equal particle number density of systems. The system particle number density is defined as the ratio of the particle number and box volume.

The influence of the annealing rate on the assembled structure, energy evolution, and cluster growth kinetics were explored at *θ* = 30° and *h*_c_ = 0.2. Three cooling rates were used including 1 × 10^5^ step per K, 2 × 10^5^ step per K and 3 × 10^5^ step per K. When 100 particles were in a 10 nm cubic box, only the cooling rates 1 × 10^5^ step per K and 2 × 10^5^ step per K were considered (Fig. 7a and b); this is because an annealing rate 2 × 10^5^ step per K has been proved to be slow enough for the formation of the final two clusters and the energy change related to the formation of each cluster was evident. The slower cooling rate (3 × 10^5^ step per K) was more useful for distinguishing the formation of the first and second clusters. However, using this cooling rate tripled the computation time (Fig. 7c and d). Thus, the moderate cooling rate of 2 × 10^5^ step per K was used for almost all simulations in this work. The larger system, which comprised 200 particles in a 12.6 nm cubic box with an equal particle number density was also tested under these conditions (Fig. 7b and c). Using the larger system did not alter the specific cluster size *N*, but it did accelerate the growth of the second cluster (red line). This demonstrates that the specific cluster sizes *N* for each particle shape is robust and independent of system's particle number. There is a sharp increase of cluster size of cluster 1 in Fig. 7d, and it is due to the contact of a partial vesicle structure and the complete vesicle cluster 1. Because the interaction of A and B beads is attractive, the partial vesicle structure (some side surfaces with A beads are exposed) can contact with the B beads of the complete vesicle (where only B beads on the bottoms of particles can be exposed). Finally, all aggregation structures should be vesicle structures with the maximum A–A contacts corresponding to the lowest energy of the system.

### 3.4 Interaction potential of two truncated cones

**Fig. 8:**
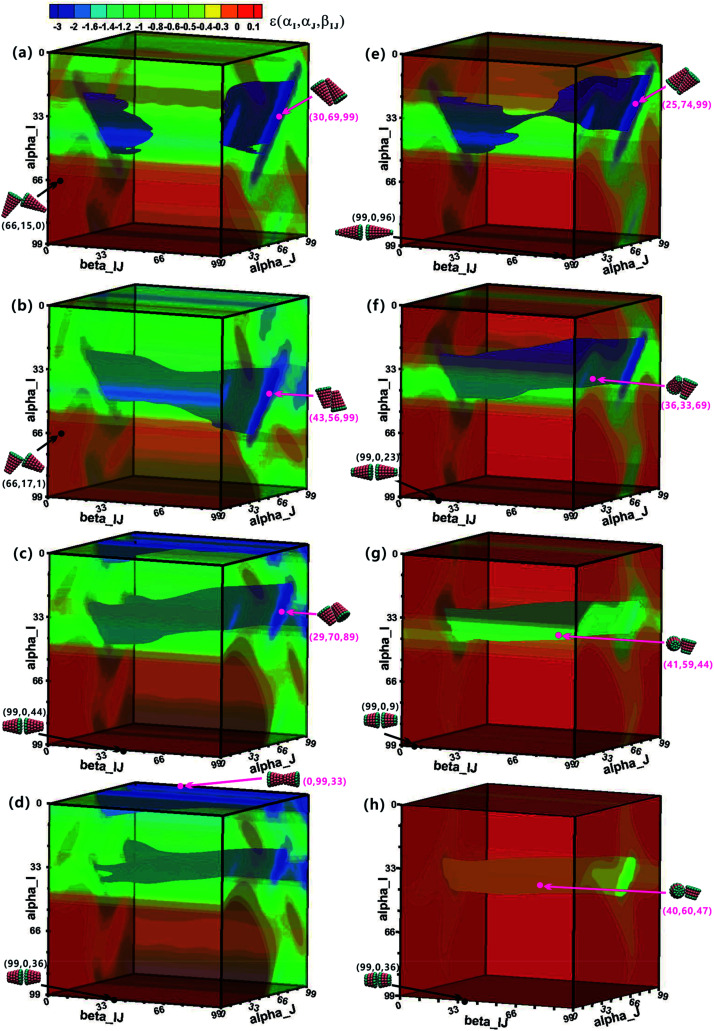
The potential energy surface (PES) of two interacting A_*x*_B_*y*_ (left column) and B_*x*_A_*y*_B_*z*_ (right column) particles. The left column (a–d) is for A_*x*_B_*y*_ particles at *θ* = 25° and *h*_c_ = 0.2, 0.4, 0.5, 0.6. The right column (e–h) is for B_*x*_A_*y*_B_*z*_ particles at *θ* = 25° and *h*_c_ = 0.2, 0.4, 0.6, 0.7. The unit of three orientation angle is π/99. The displayed colour legend in (a) is also applied in all other PESs. Pink and black points represent minimum and maximum energies in the PES, respectively, and their corresponding configurations are also shown.

To understand the assembly mechanism of various truncated cone in depth, the effect of the particle shape on the potential energy surfaces (PESs) between two interacting particles was discussed (see Fig. 8 and S5–S6† in which the colour legends are all the same to facilitate comparisons of the potential energies). In the PES data, orange and red indicate a positive potential energy, *i.e.*, two particles in these orientational zones will repulse each other. Pink points and black points indicate minimum and maximum energies on the PES, respectively, and their corresponding configurations are also shown. The orientational range in the near blue regions (lower potential energy) on the PESs is approximately the same at the same *h*_c_, but the potential energy increases with increasing *θ*. The same is true in the near red region (higher potential energy). The minimum-energy conformations of A_*x*_B_*y*_ type particles change from antiparallel side–side packing to tail–tail stacking with increasing *h*_c_ (Fig. 8), but the transformation of minimum-energy conformations of B_*x*_A_*y*_B_*z*_ type particles is different: they change from antiparallel side–side packing to orthonormal side–side packing with increasing *h*_c_ (Fig. 8 and S5–S6†). However, the final assembled vesicle structures with a lower energy have a higher packing number density (see cluster structures in Fig. 2) and they are not constructed based on these minimum-energy configurations between two particles. The minimization of the system's total energy, rather than the local energy, must be ensured at all times to encourage effective self-assembly. Self-assembly is a multi-body interaction process, and the system's total energy is the sum of the interactions of any two interacting particles. In our simulations, once a free truncated cone attempts to join the structure, it alters its orientation to find the best position, and all particles constituting the structure modify their orientations to achieve the most stable equilibrium conformation. This is akin to the local rule-based theory^43,44^ for virus capsid assembly in which a protein possesses enough local information to know where to bind to the growing cluster. To assemble into the global vesicle structure, the attractive forces between particles should be large enough to induce effective aggregation, but also weak enough to form complete vesicle structure for global energy minimization during the assembly process *via* thermal fluctuations.

## 4. Discussion

The self-assembly of truncated cones reported here generates nanoscale vesicles with a range of cluster sizes, and all formed vesicles are with similar spherical cluster geometries. The resultant unique cluster sizes and spherical cluster geometries can be explained by the particle shapes and interparticle interactions. Our study reveals that truncated cones with different shapes undergo the classical nucleation and growth pathway to form ordered structures. Thus cone-shaped particles possess ideal characteristics for self-assembly. Particles with such cone-like shape may be prepared by various experimental methods including mechanical stretching,^45,46^ soft lithography^47,48^ and phase separation.^49,50^ Mechanical stretching method can enhance particle shape diversity when using more complex starting materials,^46^ such as using Janus polymeric particles. After stretching, Janus ellipsoidal particles can be formed and subsequently half of each particle can be removed *via* selective solubilisation to obtain cone-like particle. Moreover, bullet-like structures has been created from single component particles through stretching-liquefaction method.^46^ The key advantages of the mechanical stretching method are its high throughput and precise control over particle size in three dimensions; it is also easy to scale up production through increased film thicknesses and particle loadings in the films. Beside the stretching method, soft lithography technology is also a feasible method to fabricate conical-shaped particles^47^ and, in principle, any shape particle.^48^ Surface modification can be realized using glancing angle deposition.^51–53^ Further, pinecone-shaped particles can be generated by phase separation of binary blends of block copolymer and homopolymer by varying the interfacial properties of polymers and aqueous solution; in such particles the cone side of a particle will consist of diblock copolymer.^50^ Therefore, conical particles can be produced by selectively dissolving homopolymer components. The resultant cone-shaped particle have amphiphilic top and bottom surfaces owing to the comparable interactions of the blocks with the aqueous solution. Thus, a simple fabrication method can be used to make a remarkable large variation of cone-shaped particles.

## 5. Conclusions

The self-assembly of a series of truncated cones was systematically studied by applying a BD simulation. Cones or truncated cones are important models to understand the aggregation structures of amphiphilic or surfactant-like molecules. In this work, two kinds of truncated cones (A_*x*_B_*y*_ and B_*x*_A_*y*_B_*z*_ types) with different cone angle *θ* and truncated height *h*_c_ were designed. Our simulations show that truncated cones can assemble into highly ordered nanoscale vesicles with a specific cluster size and inner interface properties. The simulations reveal that the cluster size, cluster geometry and cluster growth kinetics greatly rely on the particle shape. Cluster size is mainly determined by cone angle *θ*, while the truncated height *h*_c_ and particle types have little effect on the cluster size. The geometry of all formed clusters was spherical, as this is energetically most favourable. The effect of particle type (A_*x*_B_*y*_ or B_*x*_A_*y*_B_*z*_) on the assembly process was found to be insignificant, while the truncated height *h*_c_ has a strong influences on the cluster growth kinetics. Our results show that the truncated cones with different particle shapes undergo similar ordering pathways. Nanoscale vesicles formed from truncated cones have potential applications including serving as carrier for molecules delivery, forming a bioreactor for cell encapsulation and providing a scaffold for nanocrystals growth to grow on. The available methods to fabricate truncated cones were also discussed in this paper. This work not only provides novel insights into the self-assembly processes of nonspherical particles, but also broadens our knowledge of how to design the building unit particles for the formation of nanovesicles structures.

## Conflicts of interest

There are no conflicts to declare.

## Supplementary Material

RA-008-C8RA01100A-s001
