# Supplementary material for: Self-assembly of amphiphilic truncated cones to form hollow nanovesicles
Source: RSC Adv. 2018 Apr 10;8(24):13526–36. doi: 10.1039/c8ra01100a (PMC9079828; doi:10.1039/c8ra01100a)
Supplement: RA-008-C8RA01100A-s001 [file RA-008-C8RA01100A-s001.pdf]

Electronic Supplementary Material (ESI) for RSC Advances.
This journal is © The Royal Society of Chemistry 2018

Self-assembly of amphiphilic truncated cones to form hollow nanovesicles

Yali Wang[1] and Xuehao He[1,2,3*]

[1]*Department of Chemistry, School of science, Tianjin University, Tianjin 300350, China*

[2]*National Demonstration Center for Experimental Chemistry & Chemical engineering Education, Tianjin University, Tianjin 300350, China*

[3]*National Virtual Simulation Experimental Teaching Center for Chemistry & Chemical engineering Education，Tianjin University, Tianjin 300350, China*

Table S1. The number of *A* and *B* beads of each kind of particles and the annealing temperature range of each system with corresponding particle shape in this work.

shape	θ (°)	h_c	A_xB_y	Annealing temperature range	shape	θ (°)	h_c	$B_xA_yB_z$	Annealing temperature range
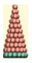	25	0.0	$A_{97}B_{26}$	270~204K					
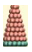	25	0.2	$A_{94}B_{26}$	255~198K	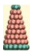	25	0.2	$B_3A_{91}B_{26}$	225~181K
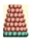	25	0.4	$A_{85}B_{26}$	220~166K	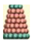	25	0.4	$B_7A_{78}B_{26}$	180~100K
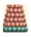	25	0.5	$A_{78}B_{26}$	220~137K	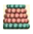	25	0.6	$B_{12}A_{56}B_{26}$	100~40K
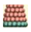	25	0.6	$A_{68}B_{26}$	190~110K	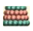	25	0.7	$B_{16}A_{40}B_{26}$	71~1K
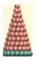	30	0.0	$A_{130}B_{36}$	300~240K					
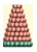	30	0.2	$A_{127}B_{36}$	290~240K	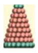	30	0.2	$B_5A_{122}B_{36}$	250~200K
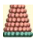	30	0.4	$A_{115}B_{36}$	260~140K	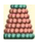	30	0.4	$B_{10}A_{105}B_{36}$	185~115K
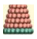	30	0.5	$A_{105}B_{36}$	250~150K	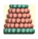	30	0.5	$B_{12}A_{93}B_{36}$	180~100K
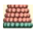	30	0.6	$A_{93}B_{36}$	230~100K	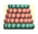	30	0.6	$B_{18}A_{75}B_{36}$	110~40K
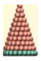	35	0.0	$A_{158}B_{47}$	300~220K					
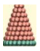	35	0.2	$A_{155}B_{47}$	290~200K	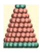	35	0.2	$B_5A_{150}B_{47}$	245~175K
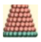	35	0.4	$A_{143}B_{47}$	270~170K	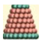	35	0.4	$B_{11}A_{132}B_{47}$	200~100K
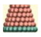	35	0.5	$A_{132}B_{47}$	260~148K	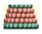	35	0.6	$B_{19}A_{97}B_{47}$	130~30K
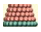	35	0.6	$A_{116}B_{47}$	230~110K	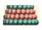	35	0.7	$B_{26}A_{71}B_{47}$	71~1K

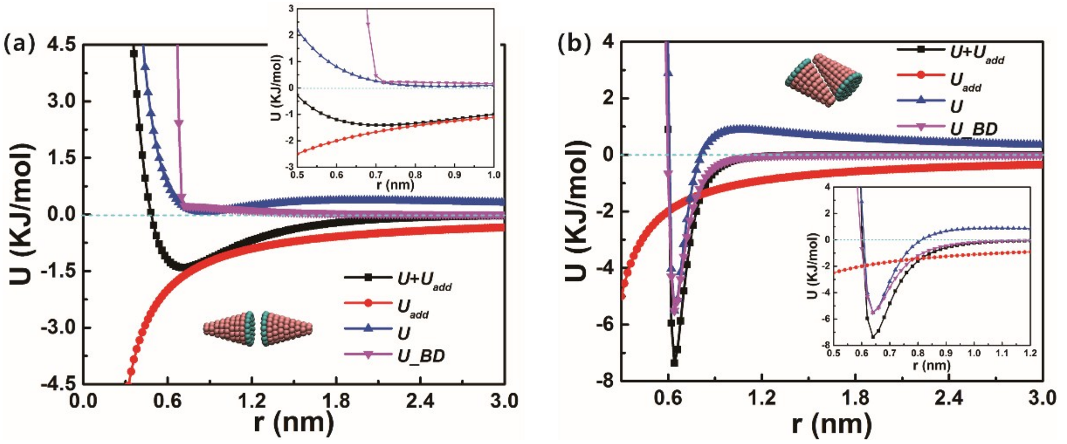

Fig. S1 The calculation of potential energy of two interacting A_xB_y particles at θ = 30° and h_c = 0.2. The orientation angles in (a) and (b) are (99, 0, 44) and (23, 76, 90), respectively.

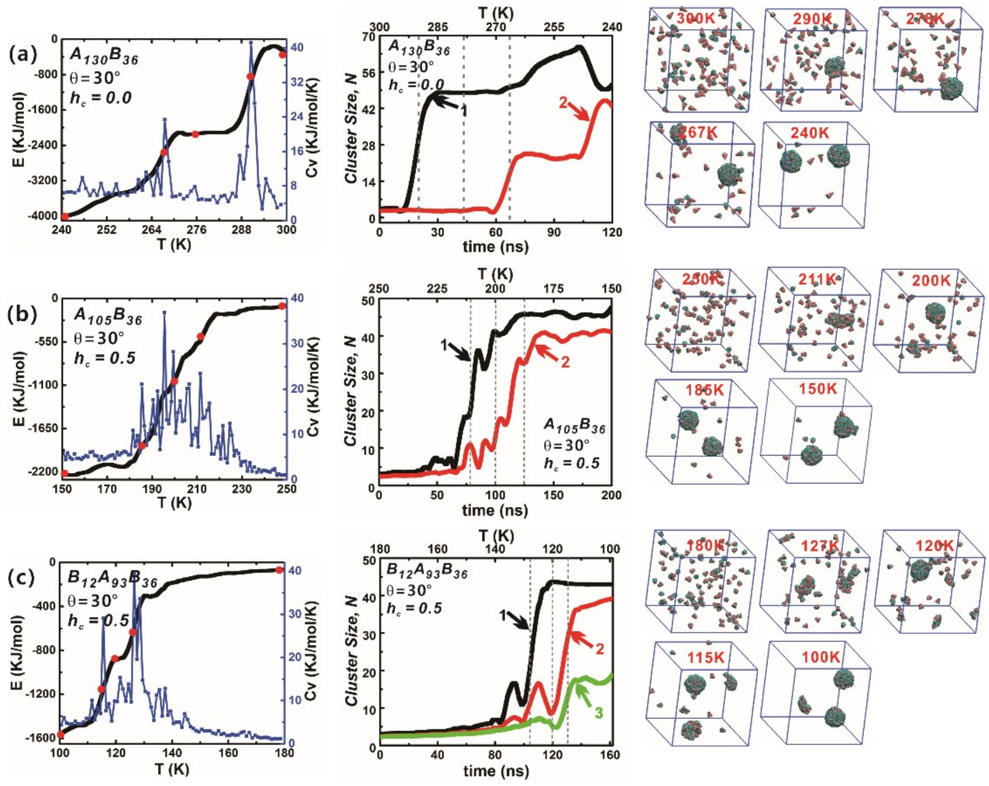

Fig. S2 The energy E, specific capacity heat *Cv* and cluster growth kinetics of A_xB_y and $B_xA_yB_z$ type particles at θ = 30° and h_c = 0.0, 0.5. The snapshots of self-assembled intermediate configurations at several typical temperatures (indicated by red points on energy evolution curves) are also shown. The specific capacity heat *Cv* reflects the system energy fluctuation in the self-assembly process. The dash lines on cluster growth kinetics correspond to those red temperature points on energy evolution curves. The final assembled structures mainly include two complete clusters, and the black, red and green curves correspond to the growth of the first, second and third cluster, respectively.

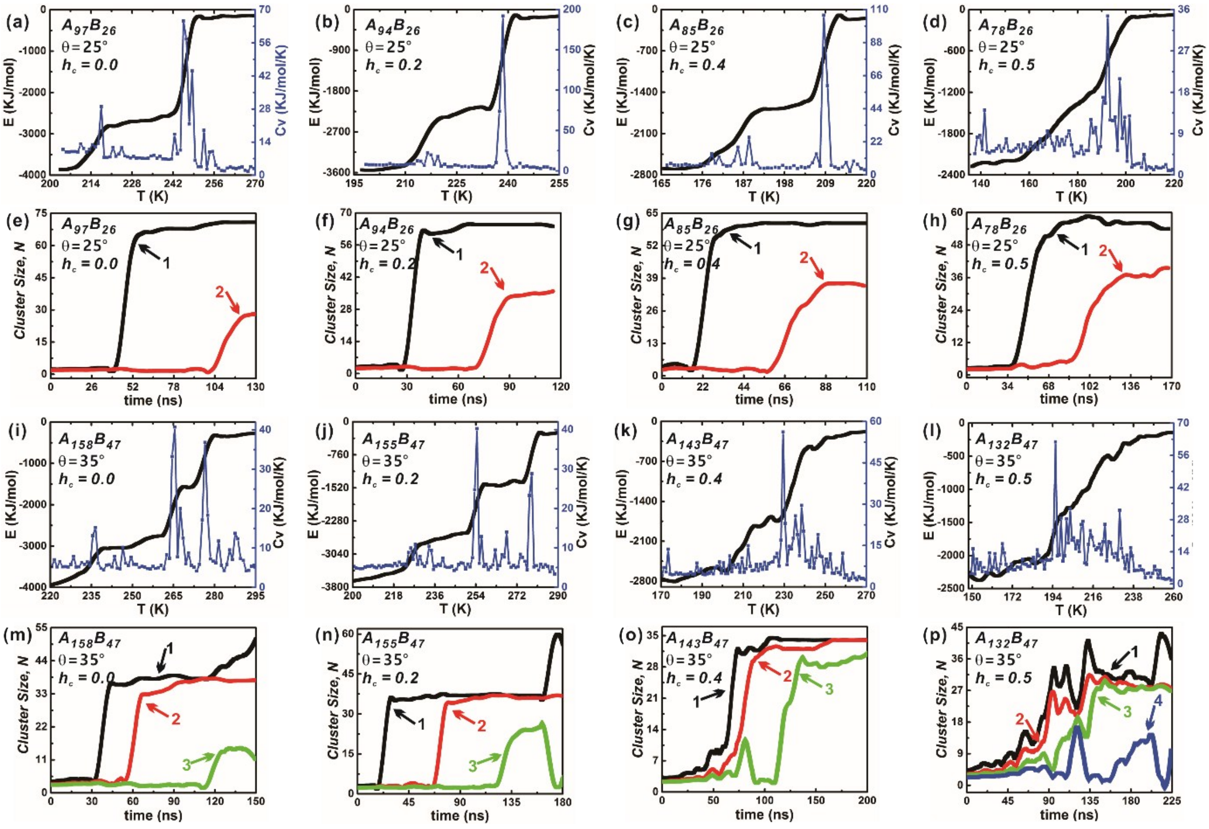

Fig. S3 Energy evolution and growth kinetics of the self-assembly of A_xB_y type particles at $\theta = 25°$ and 35°. Except for $h_c = 0.5$, the energy evolutions of A_xB_y type particles at $h_c = 0.0$, 0.2, 0.4 are like ladders. The clusters are formed gradually with one-by-one way. At $\theta = 35°$ and $h_c = 0.5$, clusters are likely to generated together, and the cluster size fluctuates rather noticeably in the assembly. The black, red, green and blue curves in (e-h) and (m-p) correspond to the growth of the first, second, third and fourth cluster, respectively.

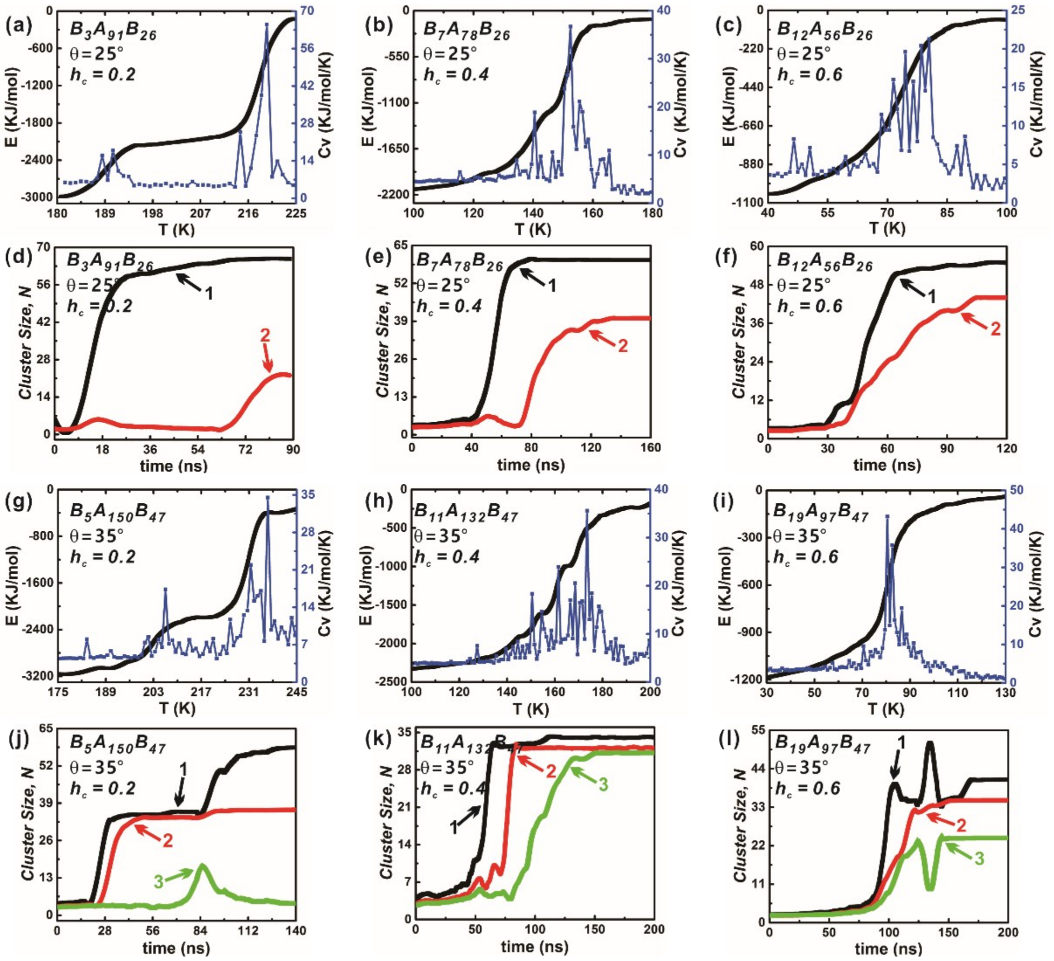

Fig. S4 Energy evolution and growth kinetics of clusters of $B_xA_yB_z$ type particles at $\theta = 25°$ and 35°. Energy decreases quickly with increasing h_c. At h_c = 0.5, several clusters begin to form simultaneously. In the case of h_c = 0.2, 0.4, clusters are formed one after another. The sharp cluster size change is due to the merger of the partial structures and complete cluster. The black, red and green curves correspond to the first, second and third cluster, respectively.

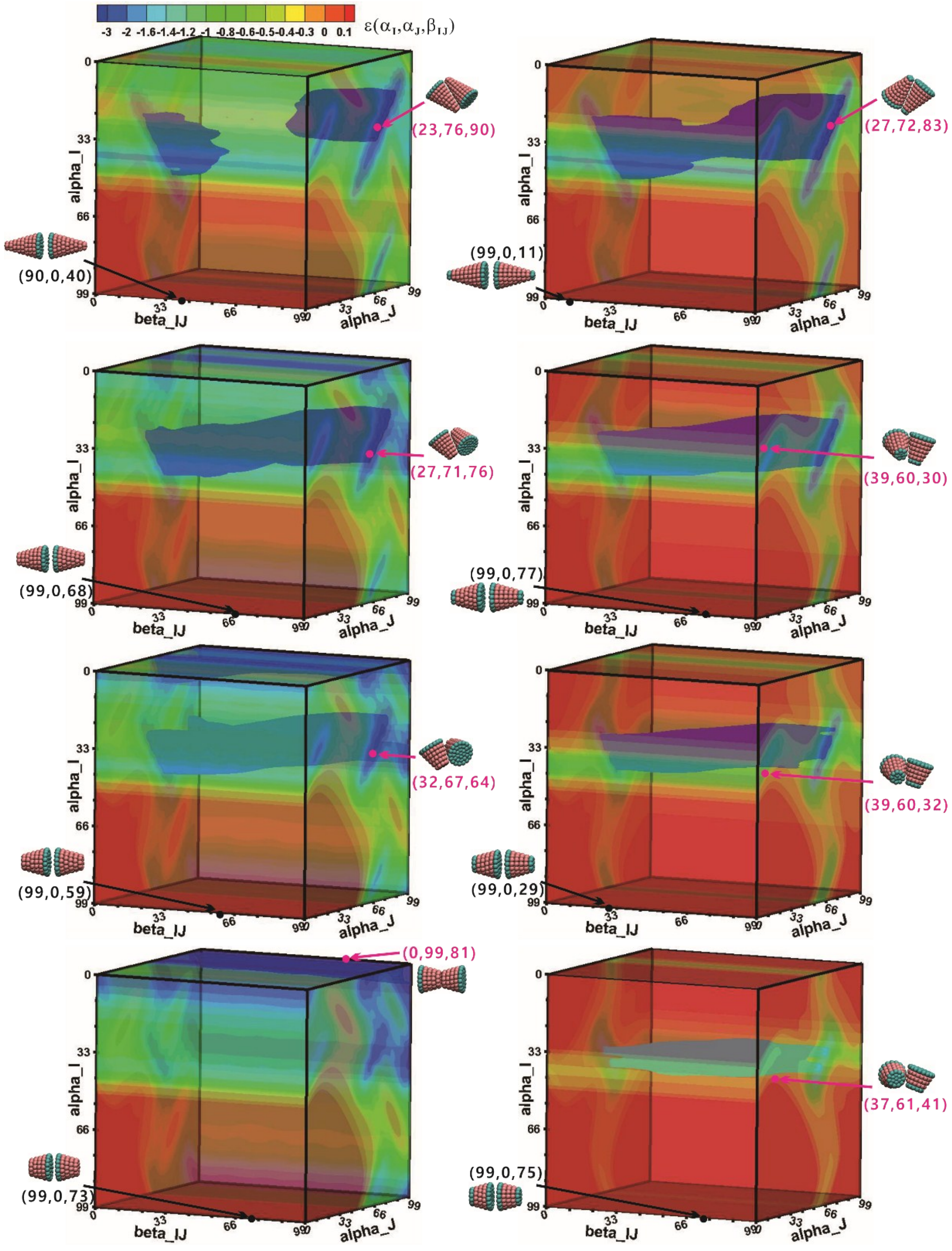

Fig. S5 The potential energy surface (PES) of two interacting A_xB_y and $B_xA_yB_z$ type particles. The left column is for A_xB_y particles at θ = 30° and h_c = 0.2, 0.4, 0.5, 0.6. The right column is for $B_xA_yB_z$ particles at θ = 30° and h_c = 0.2, 0.4, 0.5, 0.6. The unit of three orientation angle is $\pi/99$. The displayed color legend is also applied in all PESs. Pink and black points represent minimum and maximum energies on PES, respectively, and their corresponding configurations are shown.

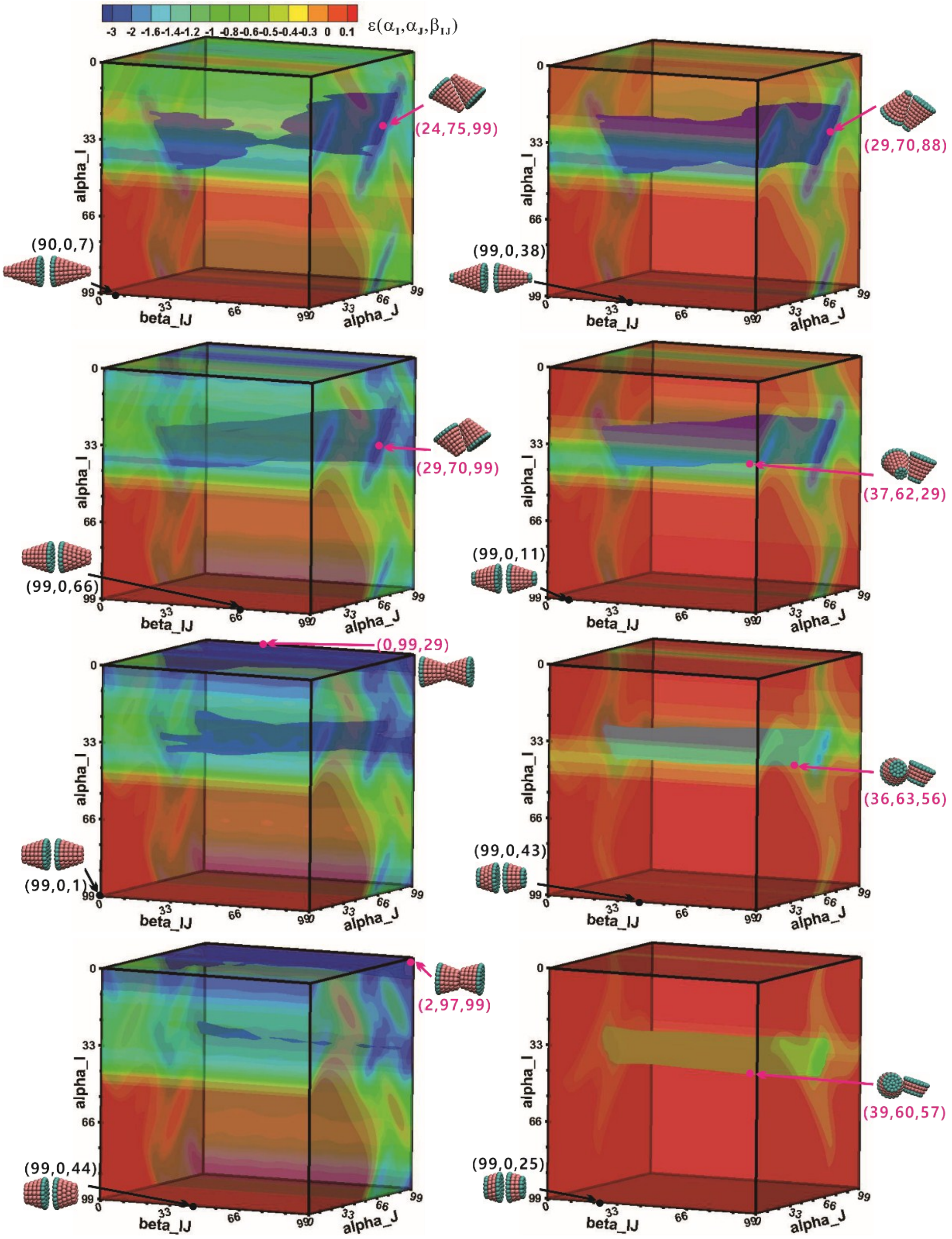

Fig. S6 The potential energy surface (PES) of two interacting A_xB_y and $B_xA_yB_z$ type particles. The left column is for A_xB_y particles at θ = 35° and h_c = 0.2, 0.4, 0.5, 0.6. The right column is for $B_xA_yB_z$ particles at θ = 35° and h_c = 0.2, 0.4, 0.6, 0.7. The unit of three orientation angle is $\pi/99$. The displayed color legend is also applied in all PESs. Pink and black points represent minimum and maximum energies on PES, respectively, and their corresponding configurations are shown.
